# Fabrication of PVDF Membranes with a PVA Layer for the Effective Removal of Volatile Organic Compounds in Semiconductor Wastewater

**DOI:** 10.3390/polym17101332

**Published:** 2025-05-14

**Authors:** Youngmin Choi, Changwoo Nam

**Affiliations:** Carbon Composites Convergence Materials Engineering, Jeonbuk National University, Jeonju 54896, Jeollabuk-do, Republic of Korea

**Keywords:** polyvinylidene fluoride (PVDF), phase-inversion membrane, dip coating, hydrophilic

## Abstract

Through the application of advanced membrane modification strategies, high-performance membranes have been developed to effectively remove organic contaminants such as toluene and xylene from wastewater. These membranes demonstrate superior antifouling resistance and long-term operational stability, offering a competitive advantage for semiconductor wastewater treatment. This study introduces a novel approach to membrane fabrication using polyvinylidene fluoride (PVDF), recognized for its cost-effectiveness and distinct antifouling properties in contaminant removal. To enhance the performance of the membrane, the solvent (DMA, DMF, NMP) that dissolves PVDF and the immersion time (30 min, 60 min, 90 min) at which phase separation occurs were identified. Additionally, the membranes were treated with polyvinyl alcohol (PVA) through multiple dip coatings to enhance their hydrophilicity before a comparative analysis was conducted. The resulting optimized membranes demonstrated high emulsion fluxes (4412 $Lm^{-2}h^{-1}{\mathrm{bar}}^{-1}$ for toluene) and achieved oil-removal efficiencies exceeding 90% when tested with various organic solvents, including toluene, cyclohexane, xylene, benzene, and chloroform. The resulting optimized membranes prove to be a reliable means of producing clean water and of efficiently separating organic contaminants from wastewater. Showcasing remarkable antifouling capabilities and suitability for repeated use without significant efficiency loss, this solution effectively addresses cost and fouling challenges, presenting it as a sustainable and efficient wastewater treatment method for the semiconductor industry.

## 1. Introduction

The rapid expansion of the semiconductor industry, driven by the increasing demand for electronics such as smartphones, home appliances, and electric vehicles, has led to a significant rise in wastewater generation during wafer-processing steps such as etching, cleaning, and deposition [1]. This wastewater often contains hazardous organic solvents [2,3] and fluorinated compounds [4,5] that pose serious environmental risks if not properly treated. In recent years, membrane-based separation has gained attention as a promising strategy for treating semiconductor wastewater due to its efficiency and scalability. Several studies have demonstrated the potential of polymeric membranes such as PVDF and PES; however, challenges remain in terms of their membrane fouling, low selectivity toward organic pollutants, and poor long-term stability under harsh chemical environments. These limitations highlight the urgent need for advanced membranes that can offer enhanced separation performance, chemical resistance, and reusability in semiconductor wastewater treatment applications.

The primary treatment methods for semiconductor wastewater include chemical, biological, and physical treatments. Chemical treatment utilizes reactions such as catalytic wet oxidation [6,7] and electrochemical oxidation [8,9]. However, this method requires complex additional processes for handling gases and other byproducts, necessitating further post-treatments. Biological treatments can decompose substances that are difficult to break down [10,11], but these processes are time-consuming and require specific environmental conditions. Physical treatment entails the separation of membranes, which facilitates the remediation of pollutants in wastewater through techniques such as adsorption and coagulation [12,13,14,15]. The use of these membranes for wastewater treatment is a cost-effective and efficient method for targeting and removing pollutants when compared with other available techniques.

To optimize the use of such membranes, parameters such as their strength, permeability, and surface characteristics need to be considered. Enhancements in performance can be achieved through techniques like plasma treatment, surface nanostructuring, and dip-coating. Plasma treatment modifies the membrane surface by exposing it to low- or high-temperature plasma, which induces both chemical and physical alterations [16,17], yet this approach is costly and struggles with uniformly treating the entire membrane. Surface nanostructuring shapes the membrane surface at the nano- or microscale, enlarging the contact area with liquids and thereby boosting performance [18,19], although it poses challenges in scalability, increased dust accumulation, and long-term maintenance. Among the various hydrophilic coatings, polyvinyl alcohol (PVA) has been applied to improve the antifouling and hydrophilic properties of PVDF membranes [20,21]. However, few studies have focused on their performance in treating semiconductor wastewater containing toxic organic solvents. This study aims to address that gap.

In this study, a membrane was fabricated for the purpose of removing organic pollutants from wastewater. This was achieved by applying a dip coat to a polyvinylidene fluoride (PVDF) membrane, followed by a polyvinyl alcohol (PVA) active layer. The PVDF support layer exhibits excellent chemical resistance and high tensile strength, rendering it an optimal choice for industrial wastewater treatment. Therefore, to fabricate the PVDF membrane, the ideal solvent for dissolving the PVDF pellets and the immersion time in deionized water required to form pores were investigated. The membrane, fabricated by dissolving PVDF pellets in N-methyl-2-pyrrolidone (NMP) and immersing them in deionized water for 60 min, was confirmed to have the most appropriate pore size. Subsequently, the optimal number of PVA-coating layers was identified, with the objective of forming a hydrophilic active layer on the PVDF support layer. The resulting membrane, designated as NMP60/PVA5, was observed to demonstrate the effective separation of water and the removal of organic contaminants from an emulsion containing 1 wt% toluene and 99 wt% water following five rounds of PVA dip-coating. To achieve robust interfacial adhesion between the PVDF support layer and the PVA active layer, an alternating dip-coating process with a 5 wt% PVA solution and a cross-linker was employed. Consequently, the membrane displayed hydrophilic and oleophobic characteristics, enabling the effective removal of organic pollutants from wastewater.

## 2. Materials and Methods

### 2.1. Materials

Polyvinylidene fluoride ($M_{w}\;=$ 530,000; 99%), Polyvinyl alcohol ($M_{w}\;=$ 89,000–98,000; 99%), and Oil Red O were purchased from Merck Co., Inc., Daemstadt, Germany. N-Methyl-2-pyrrolidone (NMP, 99%) and, dimethylacetamide (DMA, 99%) were acquired from Junsei Chemical Co., Inc., Tokyo, Japan. Span^®^80 and methylene blue trihydrate (95%) were sourced from Duksan Science Co., Ltd., Ansan, Republic of Korea. Toluene (99.8%), sodium hydroxide (98%), sulfuric acid (95%), xylene (99%), benzene (99.5%), and cyclohexane (99.5%) were procured from Samchun Chemicals Co., Ltd., Pohang, Republic of Korea. N,N-Dimethylformamide (DMF, 99.5%), glutaraldehyde (25–50 wt% aqueous solution), and chloroform (99.8%) were obtained from Daejung Chemicals & Metals Co., Ltd., Siheung, Republic of Korea.

### 2.2. Preparation of a Pristine PVDF Membrane

We conducted experiments to determine the optimal conditions for membrane fabrication, testing various solvents (DMF, DMA, NMP) and soaking times (30 min, 60 min, and 90 min) to dissolve the PVDF pellets. To identify the most suitable solvent, membranes were first fabricated using DMF, DMA, and NMP with a fixed immersion time of 60 min. The PVDF membranes were fabricated by solution-casting and nonsolvent-induced phase separation (NIPS). A 16 wt% PVDF pellet was dissolved in N,N-Dimethylformamide (DMF), dimethylacetamide (DMA), or N-Methyl-2-pyrrolidone (NMP) at 70 °C for 12 h. The resulting homogeneous solution was cast onto a glass plate and dried at room temperature for 90 min. Subsequently, the effect of immersion time (30, 60, 90 min) was investigated only for the solvent (NMP) that showed the most favorable morphology and pore structure.

### 2.3. Preparation of the NMP60/PVA Membrane

The PVDF membrane was dip-coated in a 5 wt% PVA solution for 10 min and dried at room temperature. Subsequently, it was submerged in a cross-linking solution containing 2 wt% glutaraldehyde and 0.5 wt% sulfuric acid for 5 min at room temperature and 2 min at 60 °C [22]. This process was repeated 0, 3, 5, and 7 times for each membrane, which were named NMP60/PVA0, NMP60/PVA3, NMP60/PVA5, and NMP60/PVA7. Finally, the membranes were rinsed with deionized water and dried at 60 °C.

### 2.4. Characterizations

FE-SEM (SUPRA40VP, Carl Zeiss, Oberkochen, Germany) was employed to examine the membranes’ surface morphology at a 2 kV beam voltage. Membrane thickness was measured using a digital micrometer (Mitutoyo IP65, Kawasaki, Japan). ATR-FTIR (INVENIO-S, Bruker, Billerica, MA, USA), with a range from 400 to 4000 cm^−1^, was utilized to analyze the chemical structures of the membranes. The pore diameter of the cylindrical specimens was assessed using a capillary flow porometer (CFP-1200AEL; Porous Material Inc., Ithaca, NY, USA). The water and oil contact angles and surface energy were determined using Smartdrop Plus (Femtobiomed, Inc., Seongnam, Republic of Korea). The emulsion before and after filtration was observed with an inverted microscope (Nikon Ti2-U, Tokyo, Japan).

### 2.5. Pure-Water and Emulsion Flux of the Membrane

The separation experiment between pure water and an oil-in-water emulsion was conducted using a graduated glass funnel, membrane, fritted-glass support base, clamp, triangular flask, and vacuum pump. Since gravity alone was insufficient for effective separation, the experiment was performed under vacuum at 0.8 bar to ensure accurate performance evaluation. The effective filtration area in the separation experiment was 4π cm^2^. The oil-in-water emulsion (1 wt% toluene, cyclohexane, xylene, benzene, and chloroform in water) was stabilized with Span^®^80 and subjected to 6 h of sonication to achieve uniform dispersion. The pure-water and emulsion filtration flux of each membrane was determined by the following equation:

(1) $$\mathrm{Flux}\;(Lm^{-2}h^{-1}{bar}^{-1})=\frac{V}{A\;∆t\;P}$$

where V is the permeate volume (L), A is the effective area (m^2^), ∆t is the filtration time (h), and P is the pressure (bar).

### 2.6. Flux Decline Rate of the Membranes

A 50 g weight was positioned on the membrane and moved 20 cm over the sandpaper to measure the flux of the membrane. Subsequent to measuring the flux, the decline rate of the flux was calculated using the following equation to confirm the change in the flux of the membrane:

$$\mathrm{Flux}\;\mathrm{decline}\;\mathrm{rate}=\frac{Front\;cycle\;flux}{Current\;cycle\;flux}\times 100\%$$

### 2.7. Chemical Stability of the Membranes

To ascertain the chemical stability of the membrane in alkaline, acidic, and organic solvent environments, the membrane was immersed in 0.01 M NaOH, 0.01 M H_2_SO_4_, and 1 wt% toluene emulsion for 48 h. Subsequently, the residual weight and water contact angle were assessed following a 24 h wash in deionized water.

(2) $$\mathrm{Residual}\;\mathrm{weight}\;(\%)=\frac{m_{after}}{m_{before}}\times 100$$

where m_before_ and m_after_ refer to the membrane weights (g) before and after immersion in the acid, base, and organic solvents.

### 2.8. Oil Rejection by the Membranes

The oil rejection of the emulsion filtration was determined using the following equation:

(3) $$\mathrm{Oil}\;\mathrm{rejection}\;(R,\;\%)=\left(1-\frac{C_{1}}{C_{0}}\right)\times 100\%$$

where C_0_ is the oil concentration of the emulsion before filtering, and C_1_ is the oil concentration of the emulsion after filtering, as tested by ATR-FTIR.

### 2.9. Antifouling Performance of the Membranes

The antifouling performance of the membrane was evaluated under identical conditions to those employed in the measurement of the pure-water-and emulsion fluxes. During the experimental cycle, the membrane was subjected to a washing process involving the use of ethanol. The antifouling performance was evaluated by examining the changes in the flux recovery ratio (FRR), total fouling ratio (R_t_), reversible ratio (R_r_), and irreversible fouling ratio (R_ir_) over the course of 20 cycles. The values were calculated in accordance with the following equation:

(4) $$\mathrm{Flux}\;\mathrm{recovery}\;\mathrm{ratio}\;(\mathrm{FRR},\;\%)=\frac{J_{W2}}{J_{W1}}\times 100\%$$

(5) $$\mathrm{Total}\;\mathrm{fouling}\;\mathrm{ratio}\;(R_{t},\;\%)=\left(1-\frac{J_{E1}}{J_{W1}}\right)\times 100\%$$

(6) $$\mathrm{Reversible}\;\mathrm{ratio}\;(R_{r},\;\%)=\frac{J_{W2}-J_{E1}}{J_{W1}}\times 100\%$$

(7) $$\mathrm{Irreversible}\;\mathrm{ratio}\;(R_{\mathrm{ir}},\;\%)=\left(1-\frac{J_{W2}}{J_{W1}}\right)\times 100\%$$

where J_W1_ is the initial pure-water flux ($Lm^{-2}h^{-1}{bar}^{-1}$), J_E1_ is the emulsion filtration flux ($Lm^{-2}h^{-1}{bar}^{-1}$), and J_W2_ is the second pure-water flux ($Lm^{-2}h^{-1}{bar}^{-1}$).

## 3. Results and Discussion

### 3.1. Membrane Fabrication

**Figure 1:**
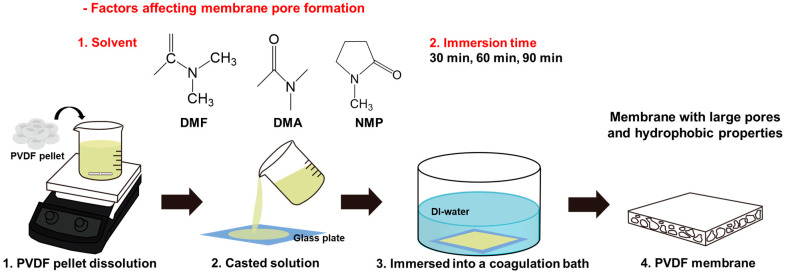
Schematic illustration of the PVDF membrane fabrication process.

When manufacturing membranes, it is important to consider factors such as strength, thickness, energy consumption, and the simplicity and convenience of the fabrication process. This study utilized straightforward nonsolvent-induced phase separation (NIPS) to fabricate the membranes for ease and convenience (Figure S1a) [23]. Factors such as membrane thickness [24], choice of solvent [25], and immersion time during the preparation of the casting solution [26] influence the porous structure and performance of the membrane. As shown in Figure 1, PVDF pellets were dissolved in a solvent and applied in a thin layer to a glass substrate. The PVDF solutions, which exhibited distinct colors with different solvents, are shown in Figure S1b. The glass substrate was then immersed in deionized water, initiating an exchange reaction between the water and the solvent, which resulted in the formation of a membrane with a porous structure. With regard to the thickness of the membrane, the fabricated membrane exhibited a thickness that was approximately 10 μm greater than that of the commercial membrane, which measured 115 μm, as shown in Figure S1c. FTIR analysis, which identifies characteristic PVDF peaks, such as CH_2_ at 1000–750 cm^−1^ and 1465 cm^−1^ and CF_2_ at 1400 cm^−1^, was conducted on both commercial and the fabricated membranes. The analysis, outlined in Figure S1d, confirmed that our membranes exhibited properties similar to those of commercial membranes [27,28]. Therefore, a membrane was produced using this method.

### 3.2. Membrane Morphology

**Figure 2:**
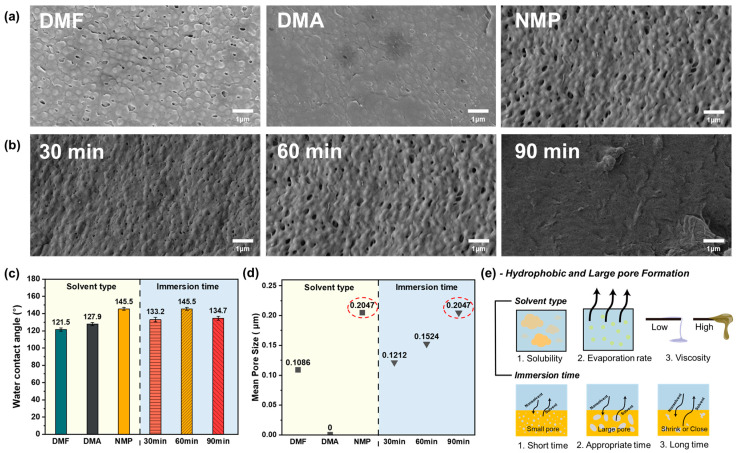
SEM images show the reverse side of the membrane produced under various conditions of (**a**) solvent type (DMF, DMA, and NMP) and (**b**) immersion time (30 min, 60 min, and 90 min). (**c**) Water contact angle measurements by solvent (DMF, DMA, and NMP) and immersion time (30 min, 60 min, and 90 min). (**d**) Mean pore size by solvent (DMF, DMA, and NMP) and immersion time (30 min, 60 min, and 90 min). (**e**) Illustration of the factors affecting pore formation depending on the solvent and immersion time.

The formation of membrane pores is influenced by both the characteristics of the solvent and the immersion time during the polymer solution preparation. To investigate this, membranes were produced using different solvents (DMF, DMA, NMP) and varying immersion times (30 min, 60 min, 90 min). To determine the most effective solvent for the membrane fabrication, the immersion time was fixed at 60 min. The optimal immersion time was identified by evaluating the performance of each solvent at this duration. Experimental conditions were based on those that showed the best results in preliminary tests, and subsequent experiments were conducted accordingly. To assess the impact of solvent and immersion time on pore formation, a detailed analysis of SEM images was performed [29,30]. As shown in Figure 2a, pore formation on the membrane surface depended on the solvent used [31]. The membrane produced with DMA showed minimal pore formation, as evidenced by the absence of visible pores. In contrast, membranes fabricated with DMF and NMP displayed pores, with the NMP membrane exhibiting a higher pore density than the DMF membrane, as confirmed by the SEM image of the opposite side (Figure S2a). The differences in pore formation can be attributed to the varying solubility and diffusion rates of each solvent and nonsolvent. This phenomenon will be discussed in more detail in a subsequent section. The NMP membrane surface displayed the highest number of pores and noticeable surface irregularity, a finding that was later supported by contact-angle and CFP analyses. Additionally, SEM images of membranes prepared with NMP as the solvent, for immersion times of 30 min, 60 min, and 90 min, were obtained and analyzed (Figure 2b and Figure S2b). Despite using the same solvent, the surface roughness and pore distribution varied with the immersion time. Notably, after 90 min of immersion, the membrane surface showed no pores, indicating that prolonged immersion does not necessarily lead to the formation of more pores. This suggests that extended immersion causes polymer chains to aggregate, forming a more compact structure that seals surface pores, resulting in a flat surface. Additionally, the membrane immersed for 30 min exhibited fewer and smaller pores compared with the membrane immersed for 60 min. This suggests that 30 min is insufficient for the complete exchange of NMP in the PVDF solution with water, leading to fewer pores. Therefore, 60 min is adequate for a proper exchange between the nonsolvent and the solvent in the PVDF solution, resulting in appropriately sized pores for wastewater treatment. A similar trend was observed in the SEM images of the opposite membrane surfaces. The initial step involved analyzing the water contact angle of the membranes produced under each condition (Figure 2c). The objective was to produce a membrane with the highest initial water contact angle, which would enable the future introduction of hydrophilicity and ensure optimal performance. The membrane produced with DMF exhibited the highest water contact angle (121.5°), followed by DMA (127.9°) and NMP (145.5°). These differences are attributed to changes in pore shape and surface roughness, which result from variations in solubility and the solution speed during the membrane-formation process. Additionally, contact-angle analysis was conducted on membranes produced with different immersion times. The membrane immersed for 60 min exhibited the highest water contact angle at 145.5°, while those immersed for 30 and 90 min had contact angles of 133.2° and 134.7°, respectively. These findings suggest that a 60 min immersion period is optimal for membrane production. To further support this conclusion, the pore size was verified through capillary flow porometry (CFP) analysis [32,33,34]. If the pores of a membrane are too small to effectively remove contaminants while allowing water to pass through, the membrane will fail to adequately filter contaminants and may also obstruct the flow of water. As shown in Figure 2d and Figure S3, the membrane prepared with DMA had a pore size below the detection limit, preventing the measurement of the mean flow pore diameter. In contrast, the mean flow pore diameters of membranes prepared with DMF and NMP were measured, with DMF showing a porosity of 0.1086 μm and NMP exhibiting the highest porosity at 0.2047 μm. As indicated in Table S1, NMP has relatively low volatility, leading to a gradual evaporation process. This contributes to slower phase separation and the formation of more uniform and smaller pores. Additionally, NMP’s higher viscosity, compared with that of DMF or DMA, results in the slower diffusion of PVDF, which facilitates the formation of more uniform pores. Furthermore, an analysis of the solubility parameters in Table S2 shows that NMP’s hydrogen bonding force (δ_h_) is lower than that of PVDF, while its dispersion force (δ_d_) and polarity force (δ_p_) are similar to those of PVDF. Although DMF and DMA are also capable of dissolving PVDF, their dissolution performance is relatively lower compared with NMP, which is likely due to the greater difference in hydrogen-bonding forces between the solvent and PVDF. Therefore, among the three solvents, NMP is the most optimal choice for dissolving PVDF. Additionally, the mean flow pore diameters of membranes prepared by immersing the PVDF solution dissolved in NMP for 30, 60, and 90 min were evaluated. The results showed mean pore diameters of 0.1212 μm, 0.1524 μm, and 0.2047 μm for the 90 min, 30 min, and 60 min immersion times, respectively. These findings highlight the significant influence of both solvent choice and immersion time on the pore formation of the membrane [35]. The phase-separation process begins rapidly but tends to slow down over time. After 30 min of immersion, phase separation occurs quickly, which may result in the formation of relatively small pores. After 60 min, the process becomes more stable, leading to the formation of larger pores. However, when immersed for 90 min, the phase separation exceeds the stabilization stage, causing pore growth to stop, and some pores may shrink or close during this stage. This can lead to a reduction in pore size as the solvent is completely removed after phase separation is completed. Additionally, prolonged immersion of PVDF may lead to excessive coagulation due to sufficient diffusion between the solvent and nonsolvent, potentially causing pore closure or the formation of closed pores. While pore growth continues for up to 60 min, the pore size may decrease during the 90 min immersion as the pore walls become thicker [36,37,38]. The immersion time in the NIPS process plays a critical role in determining the morphology and performance of PVDF membranes. Complete phase separation and coagulation require sufficient time, as the surface layer solidifies rapidly upon immediate contact with the nonsolvent. However, if the immersion time is too short (30–60 min), the internal structure may remain incompletely coagulated, leading to pore collapse or shrinkage during drying. Therefore, it is essential to extend the immersion time to at least 90 min to ensure uniform phase separation and the formation of a well-defined porous structure. On the other hand, excessively prolonged immersion can result in the overgrowth or collapse of pores due to excessive nonsolvent diffusion. Thus, optimizing the immersion time is crucial for achieving stable membrane morphology and optimal performance. By evaluating the contact angle and pore-size distribution of the membrane produced, the production process can be optimized. The membrane prepared by immersing the PVDF solution dissolved in NMP for 60 min shows optimal performance. As shown in Figure 2e, the solubility, evaporation rate, and viscosity of the solvent influence the exchange reaction rate between water and the solvent, as well as the pore structure. The immersion time is the duration during which this exchange reaction occurs, and the extent of the reaction depends on the length of this process. The results show that the membrane fabricated by immersing PVDF pellets in an NMP solution for 60 min exhibits substantial surface roughness, achieving the highest water contact angle and an optimal pore size for wastewater treatment. The degree of roughness will influence the development of superhydrophilicity when a hydrophilic coating is applied to the membrane in the future. Similarly, an optimal pore size will enhance rapid water permeation and improve the removal of organic pollutants. Among all the tested conditions, the PVDF membrane fabricated using NMP as the solvent and 60 min of immersion time exhibited the most favorable morphology and performance. This condition (NMP60) was therefore selected as the optimal baseline for subsequent coating and performance evaluation.

### 3.3. Membrane Modification

**Figure 3:**
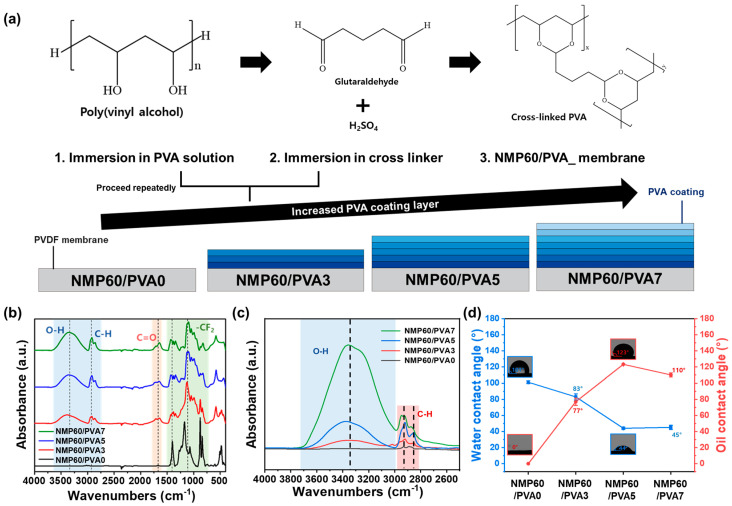
(**a**) Illustration of the process of coating PVA onto the PVDF membrane. FTIR spectra of NMP60/PVA0, NMP60/PVA3, NMP60/PVA5, and NMP60/PVA7 in the range of (**b**) 4000–2500 cm^−1^ and (**c**) 4000–2500 cm^−1^. (**d**) Water and oil contact angles for NMP60/PVA0, NMP60/PVA3, NMP60/PVA5, and NMP60/PVA7.

To apply the produced PVDF membrane to wastewater treatment, additional treatments are necessary to enhance its strength and hydrophilic and oleophilic properties. In this study, a coating process was employed to increase the membrane hydrophilicity and oleophilicity. Among the various methods for surface modification [39,40,41], dip-coating, which is a convenient and easily applicable technique, was selected for the purpose of imparting hydrophilicity [42,43]. The PVA coating, which is used to impart hydrophilicity, was applied to a membrane (NMP60), which had been previously prepared by dissolving it in NMP and immersing it for 60 min. As shown in Figure 3a and Figure S4, the membrane was coated by alternately immersing it in a 5 wt% PVA-coating solution and a mixture of 2 wt% glutaraldehyde and 0.5 wt% sulfuric acid as a cross-linking agent [44]. The purpose of immersing the membrane in a solution of glutaraldehyde and sulfuric acid following the PVA coating was to prevent the PVA coating from peeling off easily and to enhance the reusability of the membrane. Although glutaraldehyde and sulfuric acid were used to enhance membrane stability, future work will evaluate environmentally friendly cross-linking alternatives to address potential concerns such as chemical leaching and sustainability. The aldehyde functional group from glutaraldehyde reacts with the hydrogen atoms in PVA to form new cross-linking bonds on the membrane surface, while sulfuric acid strengthens these bonds. This process reinforces the PVA coating on the PVDF membrane surface, enhancing the stability and mechanical strength [45]. PVA forms acetal bonds (-C-O-C-) through a cross-linking reaction using glutaraldehyde (GA) and sulfuric acid (H_2_SO_4_). The molecular structure of the PVA attached to the membrane surface contains several hydroxyl (-OH) groups, which form hydrogen bonds with water and thereby increase the membrane’s hydrophilicity. Furthermore, the PVA coating increases the surface energy of the membrane, allowing water to easily spread on the surface. When this PVA coating is uniformly distributed on the membrane surface, water can readily contact the surface and thereby exhibit a wetting property. This makes the surface more hydrophilic. The PVA-coating process was repeated three, five, and seven times (NMP60/PVA3, NMP60/PVA5, and NMP60/PVA7) to ensure stronger adhesion and to prevent the membrane surface from peeling off easily. To verify the successful adhesion of the PVA coating and the cross-linking agent to the surface of the PVDF membrane, FTIR analysis was conducted. Initially, the FTIR spectra of each membrane and the solvents used for coating were examined. As shown in Figure S5, peaks corresponding to PVDF and crucial elements of the coating process, such as PVA and the cross-linking agent glutaraldehyde, were identified. Based on these findings, a comparison of the FTIR spectra before and after coating was performed, as shown in Figure 3b. The post-coating spectra revealed new peaks corresponding to PVA and glutaraldehyde, which were indicative of successful dip-coating. The generation of OH groups on the membrane surface improved its hydrophilicity compared with its original state, expectedly enhancing the membrane’s performance in removing contaminants from wastewater. While NMP60/PVA0 showed no noticeable peak, the O–H peak became apparent with increasing coating repetitions, and the intensity of the peak increased from NMP60/PVA3 to NMP60/PVA7 (Figure 3c). This suggests that as the number of PVA coatings rose, so did the membrane O-H content. The PVA coating determined the thickness and surface wettability of the membrane, impacting factors such as functional groups. The increase in O-H on the surface due to the PVA coating signifies that PVA that is not completely cross-linked remains on the membrane surface. The greater the number of free O-H functional groups on the membrane surface, the more hydrophilic it is, thereby increasing the water permeation rate and maintaining a high flux. Additionally, the highly hydrophilic surface exhibits increased resistance to organic contaminants, which can prevent membrane surface fouling and enhance the contaminant separation efficiency. Furthermore, the fully cross-linked PVA layer is susceptible to pore-size reduction, while the non-cross-linked PVA layer can maintain an optimal pore size, which is advantageous in selectively removing only contaminants of a specific size. To evaluate the impact of the PVA coating on wettability, the water and oil contact angles were measured. Figure 3d shows the changes in membrane wettability due to the PVA coating. Initially, NMP60/PVA0 exhibited a water contact angle of 101° and an oil contact angle of 0°, indicating hydrophobic and oleophilic properties. NMP60/PVA3 demonstrated enhanced hydrophilicity and oleophobicity, with corresponding water and oil contact angles of 83° and 77°, respectively. However, membranes with greater hydrophilic and oleophobic traits are required for effective wastewater treatment. The NMP60/PVA5 membrane featured significantly improved hydrophobic and oleophilic characteristics, with water and oil contact angles of 44° and 123°, respectively. NMP60/PVA7, which had the highest number of coatings, showed good but slightly inferior properties, with water and oil contact angles of 45° and 110°, respectively. These outcomes suggest that five PVA coatings are optimal.

### 3.4. Membrane Surface Properties

**Figure 4:**
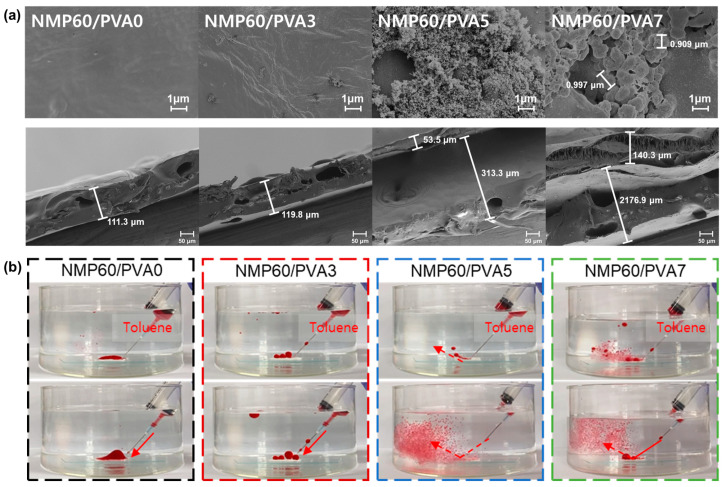
(**a**) Surface and cross-section SEM images of NMP60/PVA0, NMP60/PVA3, NMP60/PVA5, and NMP60/PVA7. (**b**) The antifouling performance of NMP60/PVA0, NMP60/PVA3, NMP60/PVA5, and NMP60/PVA7 was assessed in the presence of toluene (stained with oil red O). The red arrow indicates the direction of the toluene spill.

As the coating process advanced, morphological alterations in the membranes were discerned through scanning electron microscopy (SEM) images, exhibiting pronounced deformations in both the surface and cross-sectional views of the membranes. As shown in Figure 4a, NMP60/PVA0 displays a surface that is relatively smooth. Furthermore, while NMP60/PVA3 displays minimal alteration, the surface of the NMP60/PVA5 and subsequent membranes exhibit discernible aggregates of PVA composites. It is evident that the NMP60/PVA5 membrane exhibits a uniform distribution of small PVA composites on its surface. However, the surface of NMP60/PVA7 displays a notable accumulation of particularly large PVA composites. It has been observed that both NMP60/PVA5 and NMP60/PVA7 contain PVA composites that are present on the PVDF support surface. However, it has also been noted that the composites of NMP60/PVA7 are larger in size compared with those of NMP60/PVA5. The smaller PVA composites on the surface have a larger surface area in contact with contaminants and are less likely to be contaminated by contaminants attached to the surface. This suggests that NMP60/PVA5 achieved effective PVA coating, with the number of PVA composites increasing with each additional layer of coating. From examining the membrane cross-sectional SEM images, both the NMP60/PVA0 and NMP60/PVA3 membranes show minimal changes, retaining a single-layer PVDF structure. However, the NMP60/PVA5 and NMP60/PVA7 membranes reveal a layered structure, with the PVA coating atop the PVDF membrane. This suggests that the PVDF membrane serves as a support structure, maintaining its integrity while a PVA active layer forms on top, influencing the membrane’s hydrophilicity. It has been observed that PVA composites of less than 1 μm are formed and distributed on the surface of NMP60/PVA5, while large PVA composites of approximately 0.9 μm are distributed on the surface of NMP60/PVA7. Consequently, it can be deduced that the size of the composites increases in proportion to the increase in concentration, as evidenced by the transition from NMP60/PVA5 to NMP60/PVA7. Furthermore, the cross-sectional SEM images demonstrated the presence of well-formed pores within the membrane. Subsequent observations at the same magnification level revealed an increase in film thickness during the repeated coating process. The film thickness of NMP60/PVA0 and NMP60/PVA3 remained within the range of 111–119 μm, but starting from NMP60/PVA5, the PVDF support layer underwent expansion due to the PVA coating [46], resulting in an increase in thickness to 313.3 μm. Additionally, the presence of the PVA coating layer could be visually confirmed, starting from NMP60/PVA5. While the PVA coating of NMP60/PVA5 was 53.5 μm, it increased to 140.3 μm in NMP60/PVA7, which was approximately 87 μm thicker. This suggests that the PVA composite was agglomerated on the surface due to the multiple coatings, creating a thicker PVA layer. As shown in Figure S6, the membrane thickness exhibited a notable increase with each additional coating repetition, a finding that aligns with the observations made in the cross-sectional SEM image. While NMP60/PVA0 had a thickness of 115 μm, the thickness of NMP60/PVA3 increased to 121 μm, that of NMP60/PVA5 to 347 μm, and that of NMP60/PVA7 to 370 μm. Overall, the fabricated membranes were thicker than the commercial ones. As shown in the SEM images, the hydrophilic composite was formed on the membrane surface as a result of the PVA coating. As evidenced by the water and oil contact angle, the PVA composite on the membrane surface exerts a pronounced influence on pivotal membrane performance characteristics, including its capacity to separate organic pollutants from wastewater. To assess the antifouling capabilities of each membrane against organic pollutants, toluene was dyed with Oil Red O and dispersed in water, as depicted in Figure 4b and Videos 1, 2, 3 and 4. The behavior of the membranes was then observed and recorded. The results show that while NMP60/PVA0 can adsorb all the toluene, examination of the water and oil contact angles shows that it displays poor antifouling performance. Although NMP60/PVA3 and NMP60/PVA7 lost more toluene than NMP60/PVA0, oil droplets still adhered to the surface of the membrane. In contrast, the NMP60/PVA5 demonstrated the most effective antifouling performance against organic contaminants, as evidenced by the removal of the most toluene droplets. As observed in the SEM image, contaminants can become trapped between the large PVA composites formed on the membrane surface of NMP60/PVA7, preventing their escape. In contrast, the small PVA composites on the surface of NMP60/PVA5 can repel contaminants from the surface.

### 3.5. Membrane Performance

**Figure 5:**
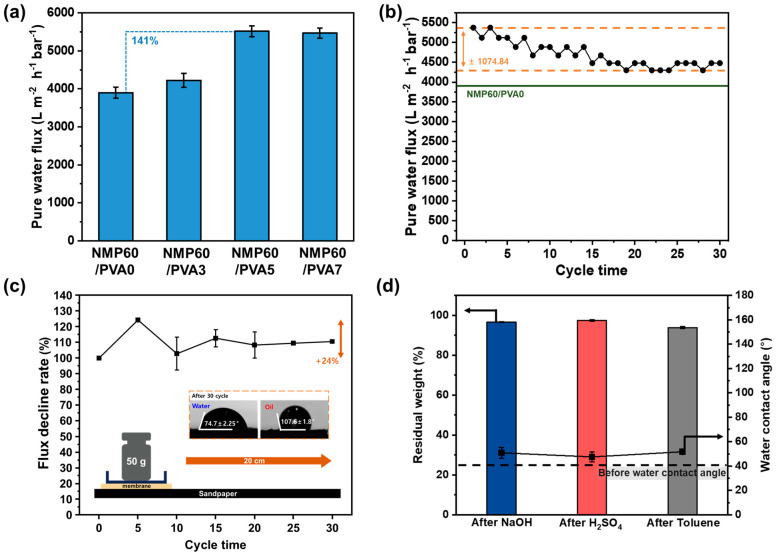
(**a**) Pure-water flux of NMP60/PVA0, NMP60/PVA3, NMP60/PVA5, and NMP60/PVA7. (**b**) Pure-water flux of NMP60/PVA5 over 30 cycles. (**c**) Flux decline rate for NMP60/PVA5 after 30 cycles on sandpaper. (**d**) Residual weight and water contact angle of NMP60/PVA5 after immersion in 0.01 M NaOH, 0.01 M H_2_SO_4_, and 1 wt% toluene for 48 h.

For membranes to be effectively utilized in an industrial setting, it is essential that they perform optimally. The wastewater matrix is composed of a diverse range of pollutants, with water representing the primary constituent. Accordingly, the membrane must demonstrate high hydrophilicity in order to selectively and rapidly permeate water. To substantiate this assertion, the pure-water flux of each membrane was quantified and presented in Figure 5a. The lowest flux was observed for NMP60/PVA0 (3899.74 $Lm^{-2}h^{-1}{\mathrm{bar}}^{-1}$), and the highest for NMP60/PVA3 (4223.19 $Lm^{-2}h^{-1}{\mathrm{bar}}^{-1}$), indicating that the PVA coating enhances hydrophilicity and thus flux performance. Moreover, NMP60/PVA5 showed a flux of 5515.63 $Lm^{-2}h^{-1}{\mathrm{bar}}^{-1}$, whereas NMP60/PVA7 showed a flux of 5468.49 $Lm^{-2}h^{-1}{\mathrm{bar}}^{-1}$. As the number of PVA-coating cycles increased, the flux initially increased; however, after a certain number of coatings, the flux exhibited a decreasing trend. This finding suggests that the thickness of the PVA-coating layer plays a pivotal role in determining membrane flux, as it directly impacts the water-transport properties. As the thickness of the PVA-coating layer increases, the flux is enhanced due to several factors, including the formation of a porous structure, improved hydrophilicity due to the swelling effect, and interfacial resistance. Due to the high hydrophilicity of PVA, the thicker the coating layer, the greater the interaction with water, which improves wettability and promotes water absorption, making water movement more efficient. The PVA-layer porous structure or swelling, which creates water channels, facilitates enhanced water permeability. However, the interfacial resistance experienced during water-molecule movement may increase in a thin PVA layer due to its interaction with the PVDF substrate. Conversely, this resistance may be alleviated in a thick coating layer, potentially contributing to an increase in flux. However, an excessively thick PVA layer can lead to an increase in the diffusion path of water molecules, which can result in an increase in resistance. Therefore, it is crucial to establish the optimal coating thickness. Consequently, it was determined that NMP60/PVA5, which demonstrated the highest hydrophilicity and flux, had the most suitable coating number. A series of 30 cycles were performed to evaluate the long-term efficacy of the membrane (Figure 5b), demonstrating a reduction in flux from 5374.203 $Lm^{-2}h^{-1}{\mathrm{bar}}^{-1}$ to 4478.503 $Lm^{-2}h^{-1}{\mathrm{bar}}^{-1}$ after 30 cycles, yet the value remained higher than that of NMP60/PVA0, indicating that PVA is securely bonded to the membrane surface after 30 cycles. For effective application in wastewater treatment, it is crucial that the membrane or coating does not tear or peel off under various external forces. The resilience of the NMP60/PVA5 against external forces was assessed by mounting it on sandpaper, placing a 50 g weight on it, and pulling it 20 cm through 30 cycles, as shown in Figure 5c. During these cycles, the flux initially showed an increase of about 24%, then stabilized. Moreover, after 30 cycles, the water and oil contact angles were measured at 74° and 107°, respectively, indicating that the hydrophilic and oleophobic properties were preserved. Also, as depicted in Figure S7a, the membrane surface was undamaged and exhibited no signs of tearing. Microscopic examination revealed a combination of smooth and rough areas due to the contact with sandpaper. Additionally, the FTIR analysis confirmed that PVA and the cross-linking agent remained intact, as did the O-H, C-H, and C=O functional groups (Figure S7b). This confirms the durability of the PVA coating, despite external forces. Moreover, industrial wastewater sources, including from the semiconductor and textile industries, contain a range of acidic, alkaline, and organic pollutants. Consequently, in order for the membrane to be deemed suitable for wastewater treatment, it is essential that it exhibits considerable resilience to these challenging conditions. This was tested by immersing the membrane in a 0.01 M solution of NaOH and H_2_SO_4_, as well as a 1 wt% toluene solution (Figure 5d). Following immersion, the original weight was retained in the NaOH and H_2_SO_4_ solution at a rate of over 95%, and in the toluene solution at a rate of 93%. The water contact angles in these solutions were 50°, 47°, and 51°, respectively, thereby confirming the maintenance of hydrophilic properties. Lastly, an FTIR analysis was conducted to precisely assess the PVA coating. The results of this analysis are displayed in Figure S8 and confirm that the PVA coating was well preserved. It can be concluded that the NMP60/PVA5 membrane exhibits notable chemical resistance to acidic, alkaline, and organic environments. This is attributed to the distinctive characteristics of PVDF pellets and the robust adhesion of PVA, which is facilitated by the cross-linker.

### 3.6. Oil/Water Separation by the Membrane

**Figure 6:**
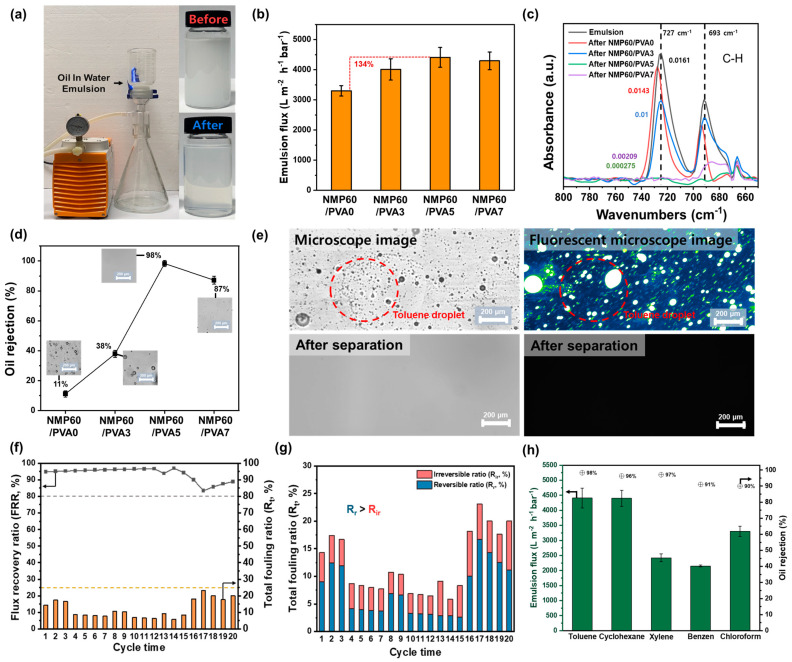
(**a**) Photographs of the emulsion separation experiment. (**b**) Emulsion flux of 1 wt% toluene emulsions using NMP60/PVA0, NMP60/PVA3, NMP60/PVA5, and NMP60/PVA7. (**c**) FTIR spectra in the range of 800–650 cm^−1^ before and after separation experiment of 1 wt% toluene emulsion using NMP60/PVA0, NMP60/PVA3, NMP60/PVA5, and NMP60/PVA7. (**d**) Oil rejection by NMP60/PVA0, NMP60/PVA3, NMP60/PVA5, and NMP60/PVA7. (**e**) Microscopic and fluorescence microscopic images after separation experiments of 1 wt% toluene emulsions using NMP60/PVA5. (**f**) Flux recovery rate (FRR) and total fouling rate (R_t_) over 20 cycles for a 1 wt% toluene emulsion. (**g**) Reversible ratio (R_r_) and irreversible fouling ratio (R_ir_) from the total fouling ratio (R_t_) over 20 cycles. (**h**) Emulsion fluxes and oil rejection by the NMP60/PVA5 for 1 wt% emulsions of toluene, cyclohexane, xylene, benzene, and chloroform.

The optimal membrane is an NMP60/PVA5 separator manufactured by dissolving PVDF in NMP, casting the film onto a glass plate, submerging it in water for 1 h, and then dip-coating it in a PVA solution with a cross-linking agent, with this process being repeated five times. To assess the viability of the membrane for real-world wastewater treatment applications, we conducted experiments to evaluate its capacity to separate water and organic matter. Separation experiments utilized 200 mL of blue-dyed water and 200 mL of red-dyed toluene, as shown in Figure S9. The NMP60/PVA5 membrane required 9 min to filter 200 mL of water, and subsequently, toluene did not permeate through, confirming that the membrane effectively separates water and organic substances. Comparative separation performance between the NMP60/PVA0 and NMP60/PVA5 membranes is documented in Videos 5 and 6. NMP60/PVA0 allowed the passage of both water and toluene, demonstrating that the PVA coating improves the membrane liquid–liquid separation capabilities. Upon examination after the experiment, NMP60/PVA5 remained intact, and its surface was stained blue by the water, indicating its durability under pressure during wastewater treatment. The separation experiment was conducted using a 0.8 bar vacuum pump, as shown in Figure 6a. Additionally, as illustrated in the accompanying image, the emulsion was initially turbid due to suspended oil droplets. However, it was confirmed that the oil droplets were effectively removed, resulting in a transparent solution following the membrane treatment. The results demonstrated that the separation experiment was conducted successfully under the specified experimental settings. To evaluate the suitability of NMP60/PVA5 for industrial wastewater treatment, the flux was measured in separation experiments using an emulsion composed of a 1 wt% toluene and 99 wt% water solution (Figure 6b). Initially, the emulsion fluxes were quantified, yielding the following values: NMP60/PVA0 (3303.12 $Lm^{-2}h^{-1}{\mathrm{bar}}^{-1}$), NMP60/PVA3 (4011.93 $Lm^{-2}h^{-1}{\mathrm{bar}}^{-1}$), NMP60/PVA5 (4412.16 $Lm^{-2}h^{-1}{\mathrm{bar}}^{-1}$), and NMP60/PVA7 (4297.02 $Lm^{-2}h^{-1}{\mathrm{bar}}^{-1}$). The NMP60/PVA5 combination exhibited the highest flux, representing a 134% increase compared with that of the NMP60/PVA0 system. Furthermore, FTIR analysis was conducted on the emulsion following the separation experiment to ascertain the efficacy of the membrane in removing organic pollutants (Figure 6c). The C-H peak of toluene remains in NMP60/PVA0, NMP60/PVA3, and NMP60/PVA7, while it is completely removed in NMP60/PVA5. Furthermore, the C-H peaks at 727 cm^−1^ and 693 cm^−1^ were subjected to a more comprehensive analysis to substantiate the presence of toluene. As a consequence, the NMP60/PVA0 and NMP60/PVA3 samples exhibited distinct peaks, whereas the NMP60/PVA5 and NMP60/PVA7 samples displayed insignificant peaks. This suggests that the removal efficacy of toluene in the emulsion is enhanced with an increase in the number of PVA coatings. Additionally, a visual change in the emulsion following the separation experiment was observed. In addition, the oil-removal rate was confirmed by the height difference of the characteristic 727 cm^−1^ peak of toluene in Figure 6d [47]. Consequently, NMP60/PVA0 exhibited minimal removal rates of 11%, while NMP60/PVA3 demonstrated a considerably higher rate of 37%. In contrast, NMP60/PVA7 displayed a noteworthy removal rate of 87%, although it did not reach the level of excellence observed in the other membranes. NMP60/PVA5, which exhibited the most favorable hydrophilicity and oleophobicity, demonstrated the highest removal rate of 98%. Furthermore, as shown in the images included in the graph and in the microscopic images presented in Figure S10, residual oil droplets were observed in the emulsions treated with NMP60/PVA0, NMP60/PVA3, and NMP60/PVA7. In contrast, the emulsion treated with NMP60/PVA5 demonstrated the complete disappearance of all oil droplets. Therefore, the transparency observed in the NMP60/PVA5 emulsion was found to correlate with the removal efficiency, indicating that this method may be an effective approach for the removal of small organic contaminant droplets. To further substantiate these findings, the pollutant toluene was fluorescently labeled, emulsions were prepared, and separation experiments were conducted using NMP60/PVA5 (Figure 6e). Following the completion of the separation experiments, the emulsions were subjected to examination under a fluorescence microscope. This revealed that a significant quantity of pollutants was present within the emulsion prior to separation, yet no evidence of such pollutants could be discerned following the procedure. These findings substantiate the efficacy of the hydrophilically modified NMP60/PVA5 in the removal of organic pollutants from industrial wastewater. It is imperative that membranes utilized for wastewater treatment demonstrate optimal separation efficiency, even when subjected to the presence of finely dispersed pollutants [48]. Additionally, changes in the FRR, R_t_, R_r_, and R_ir_ for a 1 wt% toluene emulsion of NMP60/PVA5 were measured over 20 cycles. Consequently, as shown in Figure 6f, the FRR remained above 80% for 20 cycles, while the R_t_ value stayed below 25%. These findings highlight the excellent antifouling capabilities of NMP60/PVA5 against toluene, enabling its prolonged reuse. The ratios of reversible and irreversible components of the R_t_ value were analyzed (Figure 6g). With a fouling ratio under 25%, the R_r_ was higher than the R_ir_. This suggests that a significant portion of the fouling can be removed by washing, thereby maintaining membrane performance. Notably, R_r_ primarily indicates surface fouling, while R_ir_ results from chemical bonding or deep contaminant penetration, inferring that NMP60/PVA5 predominantly causes surface fouling. Hence, NMP60/PVA5 can be effectively maintained through regular cleaning, enhancing the efficiency of the semiconductor wastewater treatment system. As demonstrated in Figure 6h, wastewater typically contains a higher proportion of water than contaminants. The NMP60/PVA5 membrane effectively prevented the passage of organic pollutants, such as toluene, cyclohexane, xylene, benzene, and chloroform, which are finely dispersed in water, allowing only water to pass through. This action facilitates the removal of harmful organic pollutants from wastewater upon discharge. The variation in flux for each emulsion can be attributed to the properties of the contaminant, including its density. The fluxes for each 1 wt% emulsion were recorded as 4412 $Lm^{-2}h^{-1}{\mathrm{bar}}^{-1}$ for toluene, 4402 $Lm^{-2}h^{-1}{\mathrm{bar}}^{-1}\;$ for cyclohexane, 2423 $Lm^{-2}h^{-1}{\mathrm{bar}}^{-1}$ for xylene, 2150 $Lm^{-2}h^{-1}{\mathrm{bar}}^{-1}$ for benzene, and 3303 $Lm^{-2}h^{-1}{\mathrm{bar}}^{-1}$ for chloroform. Furthermore, the removal rate for each emulsion was calculated. For five emulsions, the oil removal rate was over 90%, with toluene at 98%, cyclohexane at 96%, xylene at 97%, benzene at 91%, and chloroform at 90%. Furthermore, following the execution of a separation experiment for each emulsion and the conduction of FTIR analysis, it was ascertained that only the O-H peak was manifested (Figure S11a). Additionally, the emulsion had become transparent upon visual confirmation (Figure S11b). These results demonstrate that NMP60/PVA5 exhibits excellent flux and removal performance in treating wastewater containing a wide range of organic pollutants. In addition, the performance of the fabricated membrane was evaluated by comparing its pure-water flux, emulsion flux, and oil-removal efficiency with those of previously reported membranes (Table 1). The results demonstrated that the NMP60/PVA5 membrane exhibited superior pure-water flux and emulsion flux compared with conventional membranes, along with a high oil-removal efficiency of 98.3%. The collective findings of this study indicate that the NMP60/PVA5 membrane possesses excellent filtration performance and high contaminant removal efficiency, thereby underscoring its potential for effective water-treatment applications.

## 4. Conclusions

In order to remove organic pollutants from wastewater, the conditions for membrane fabrication were optimized, with a particular focus on solvent types and immersion durations in the context of PVDF, which is known for its excellent chemical resistance. Specifically, the membrane preparation method, which involved NMP dissolution and a 60 min immersion period, exhibited optimal performance. Subsequently, the hydrophilic characteristics of the membrane were augmented through dip-coating with a PVA active layer, thereby facilitating the efficient elimination of pollutants. The NMP60/PVA5 material, which underwent five cycles of dip-coating with PVA, exhibited hydrophilic and oleophobic characteristics, as evidenced by a water contact angle of 44° and an oil contact angle of 123°. These properties contributed to high flux rates against a variety of organic pollutants, with a flux recovery rate exceeding 80% and a contamination rate below 25% over 20 cycles. Furthermore, emulsion fluxes produced with diverse organic solvents, including toluene, cyclohexane, xylene, benzene, and chloroform, exhibited elevated values and demonstrated oil-removal rates exceeding 90%. To ensure practical applicability, however, future studies should further evaluate the scalability, cost-effectiveness, and environmental sustainability of this membrane fabrication method, especially in comparison to existing commercial technologies.

## Figures and Tables

**Table 1:** Performance comparison table with other membranes.

Membrane	Pure Water Flux (L m^−2^ h^−1^ bar^−1^)	Emulsion Type	Emulsion Flux (L m^−2^ h^−1^ bar^−1^)	Oil Rejection (%)	Ref.
M40	-	1 wt% hexadecane	90.0	98.0	[49]
ZQDs/CA	581.3	1 wt% diesel	350.5	98.0	[50]
m-PVDF-2.5	~3700	1 wt% n-dodecane	690	99.5	[51]
TA-Ti-5@PVDF	7643.5	1 wt% toluene	802.5	98.5	[52]
PPS/TA-PEI/β-FeOOH-15	~6200	1 wt% hexane	3770.9	98.1	[53]
NMP60/PVA5	5515.6	1 wt% toluene	4412.16	98.3	***This work***

## Data Availability

The original contributions presented in this study are included in the article/Supplementary Materials. Further inquiries can be directed to the corresponding author.

## Supplementary Materials

The following supporting information can be downloaded at: https://www.mdpi.com/article/10.3390/polym17101332/s1.
