# Supplementary material for: Fabrication of PVDF Membranes with a PVA Layer for the Effective Removal of Volatile Organic Compounds in Semiconductor Wastewater
Source: Polymers (Basel). 2025 May 14;17(10):1332. doi: 10.3390/polym17101332 (PMC12114977; doi:10.3390/polym17101332)
Supplement: Supplementary file 1 [file polymers-17-01332-s001.zip › polymers-3626479-supplementary.pdf]

Supporting Information

Fabrication of PVDF Membranes with a PVA Layer for the Effective Removal of Volatile Organic Compounds in Semiconductor Wastewater

Youngmin Choi and Changwoo Nam *

Carbon Composites Convergence Materials Engineering, Jeonbuk National University, Jeonju 54896, Jeollabuk-do, Republic of Korea
* Correspondence: cun120@jbnu.ac.kr

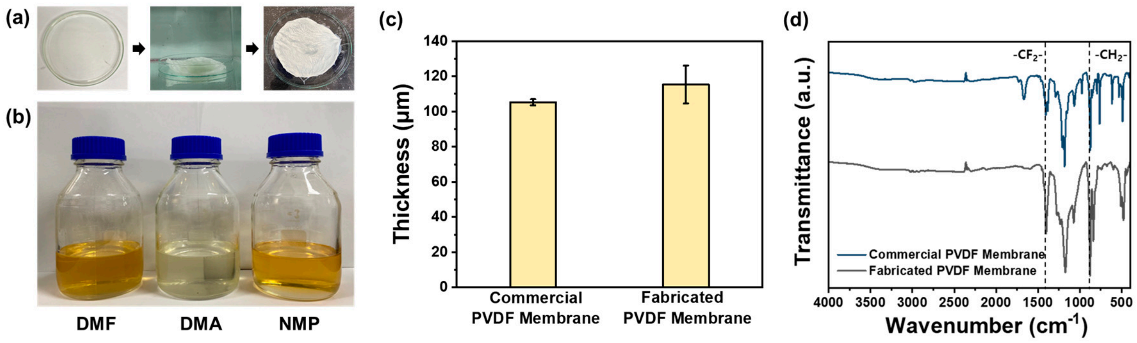

Figure. S1. (a) Membrane production process through phase separation. (b) Colors of PVDF solutions produced using DMF, DMA, and NMP. Comparison of (c) thickness and (d) FTIR spectra of commercial and fabricated membranes.

Solvent	DMF	DMA	NMP
Structural formula	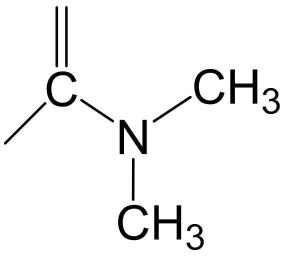	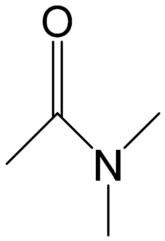	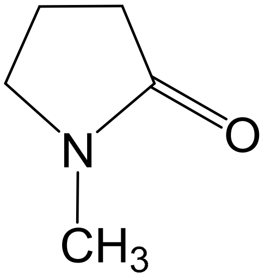
Chemical formula	C_3H_7NO	$C_4H_9N_2O$	$C_5H_9N_2O$
Solubility in water	Miscible	Miscible	Soluble
Density (g/mL)	0.948	0.937	1.028
Vapor pressure (Pa at 20 °C)	516	300	38
Viscosity (mPa·s at 20 °C)	0.92	0.945	1.661

Table. S1. Table of Solvent (DMF, DMA, and NMP) Properties.

	δ_d (M Pa$^{1/2}$)	δ_p (M Pa$^{1/2}$)	δ_h(M Pa$^{1/2}$)
PVDF	17.1	12.6	10.6
DMF	17.4	13.7	11.3
DMA	16.8	11.5	10.2
NMP	18	12.3	7.2

Table. S2. Table of solubility parameter of PVDF and each solvent (DMF, DMA, and NMP).

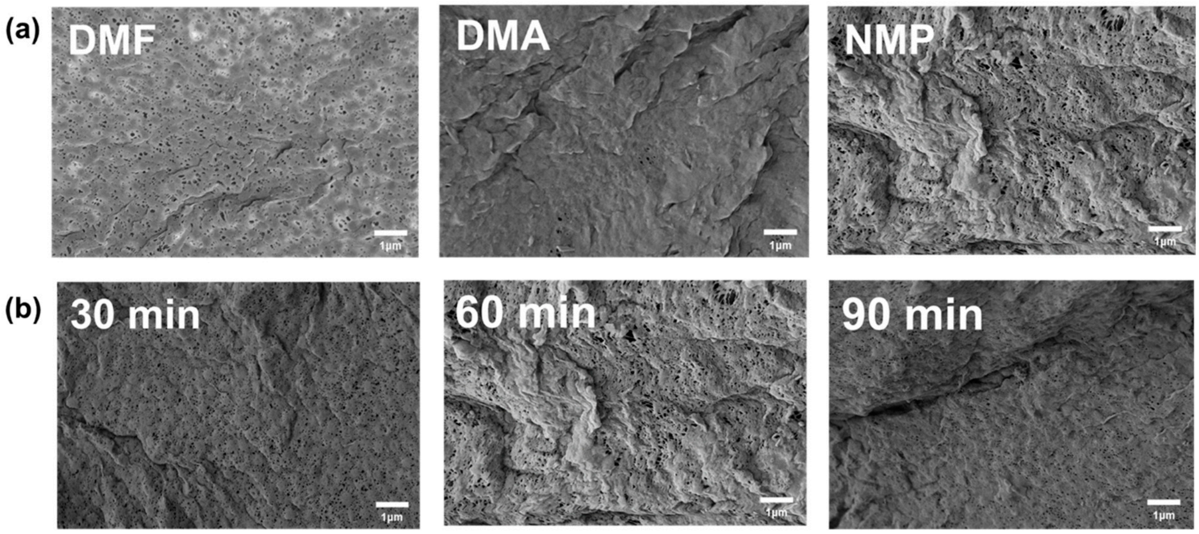

Figure. S2. SEM images of the front of the membrane fabricated under various conditions: (a) using different solvents (DMF, DMA, and NMP) and (b) with varying immersion times (30 min, 60 min, and 90 min).

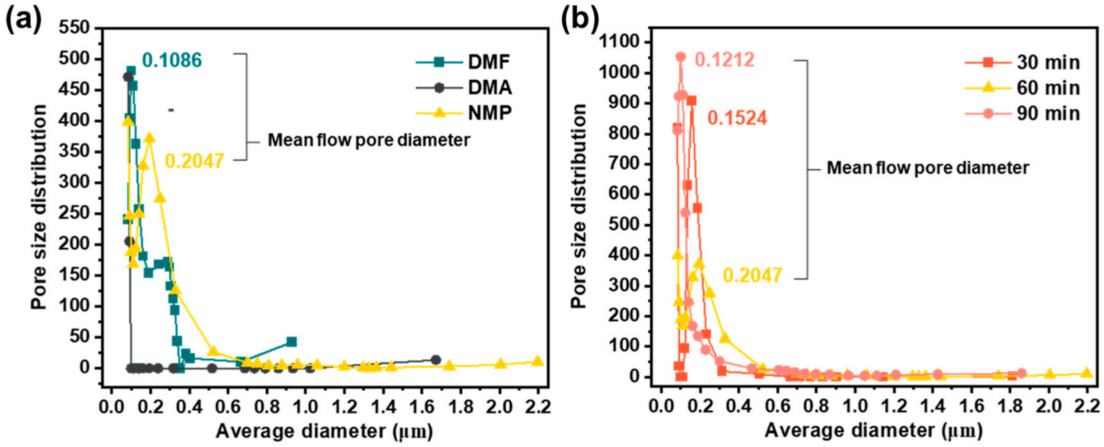

Figure. S3. Pore size distribution by (a) solvent (DMF, DMA, and NMP) and (b) immersion time (30 min, 60 min, and 90 min)

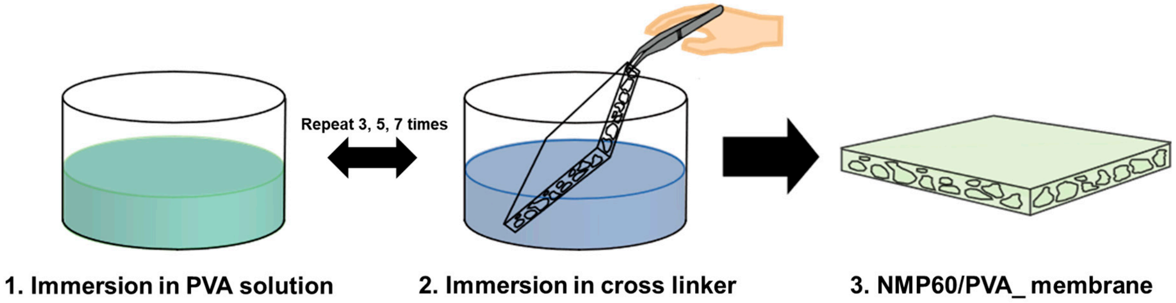

Figure. S4. Illustration depicts the process of coating PVA on a PVDF membrane.

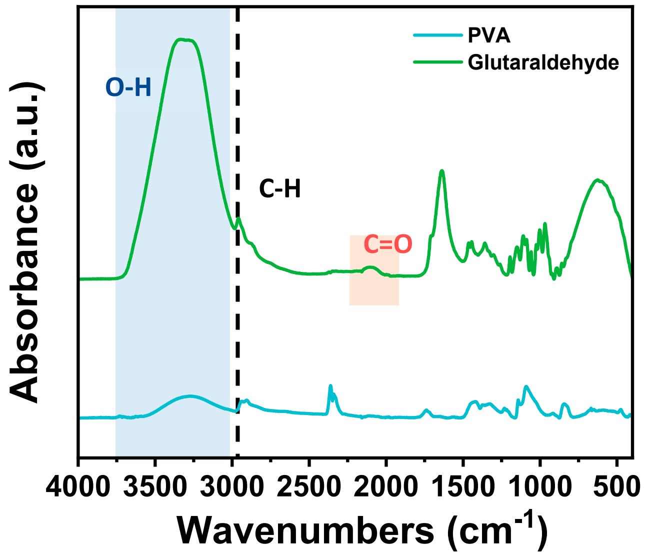

Figure. S5. FTIR spectra of PVA and glutaraldehyde.

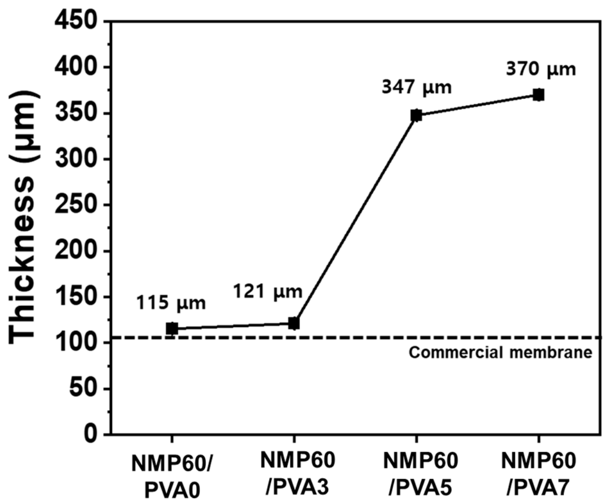

Figure. S6. Thickness of NMP60/PVA0, NMP60/PVA3, NMP60/PVA5, and NMP60/PVA7.

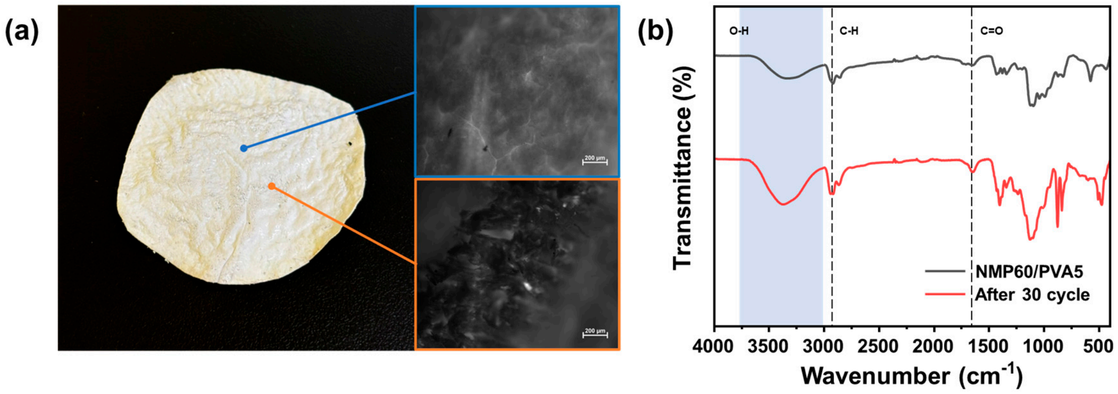

Figure. S7. (a) Digital and microscopic images and (b) FT-IR graph of the NMP60/PVA5 before and after 30 cycles.

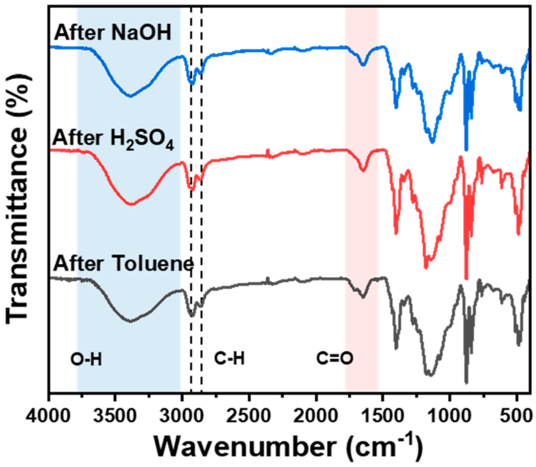

Figure. S8. FT-IR graph of the NMP60/PVA5 surface after 48 h immersion in NaOH, H_2SO_4 and toluene.

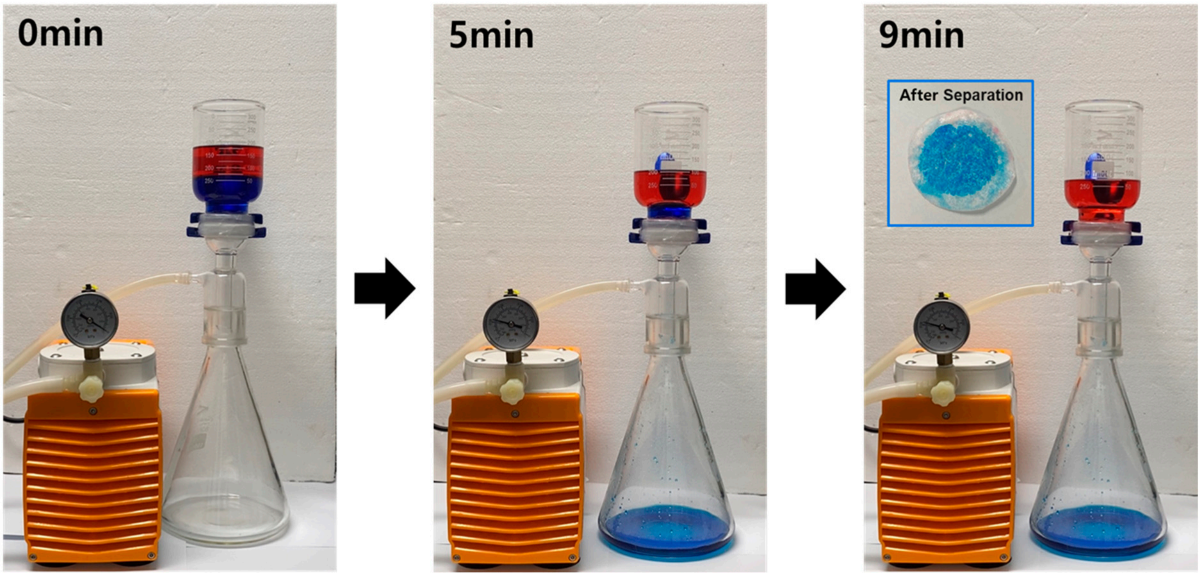

Figure. S9. Digital image of a separation experiment (NMP60/PVA5) involving 200 ml of water (stained with methylene blue) and 200 ml of toluene (stained with Oil red O).

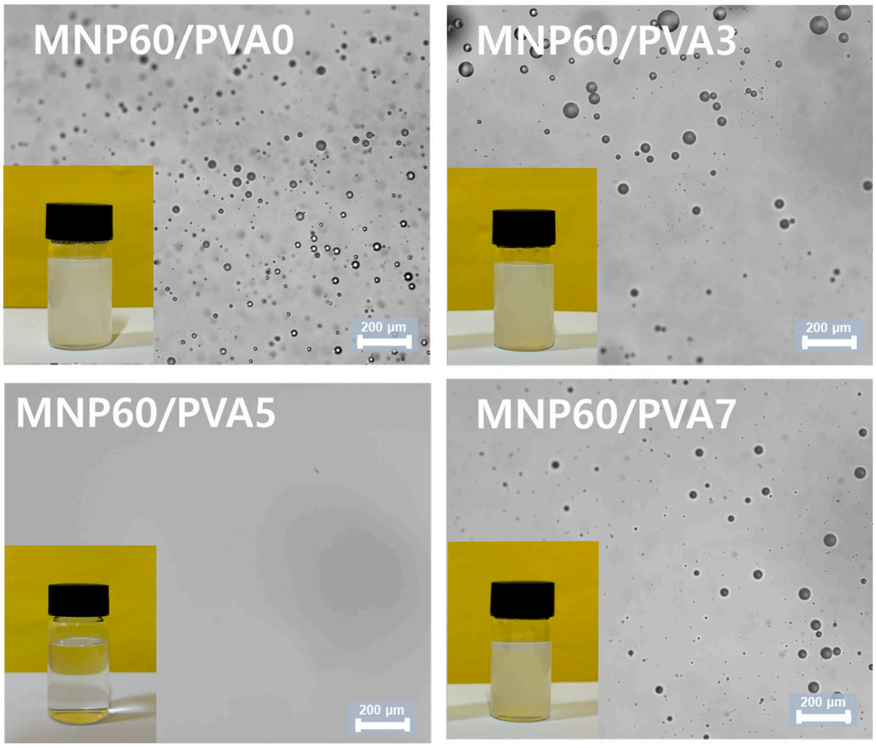

Figure. S10. Photograph and microscopic images after separation experiments of 1 wt% toluene emulsions using NMP60/PVA0, NMP60/PVA3, NMP60/PVA5, and NMP60/PVA7.

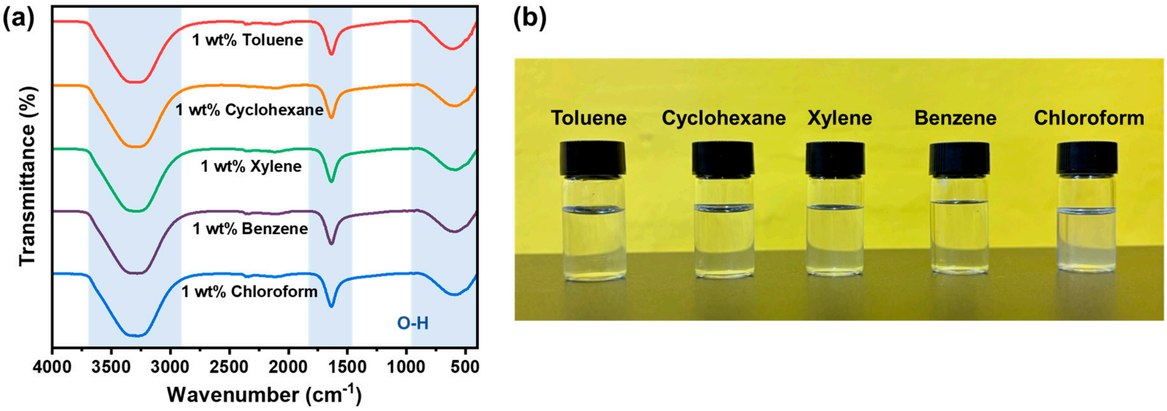

Figure. S11. (a) FT-IR spectra and (b) photograph after separation experiments of the NMP60/PVA5 for 1wt% emulsions of toluene, cyclohexane, xylene, benzene, and chloroform.
